# Abuse of Alcohol in Medical Practice

**Published:** 1868-01

**Authors:** 


### ABUSE OF ALCOHOL IN MEDICAL PRACTICE.

We are rejoiced to witness among the leading physicians in England, a convention of the great evils flowing from the indiscriminate use of alcholic stimulants in diseases. A lecture by Samuel Wilks, M.D., delivered at Guy's Hospital, appears in the *London Lancet* for July. We quote from it the following paragraph: "I may remind you of what you yourselves have witnessed—that fevers will do well without this remedy. So wedded, however, are some to the idea of the absolute necessity of stimulant, that they have expressed almost incredulity when they have heard it stated that fever will terminate favorable without them. Of course, stimulants are often needed; but young persons with typhus and typhoid do far better, I believe, without them. That they make good recoveries on simple milk diet is a fact which my hospital cases prove, and which no argument can gainsay; and on the other hand I have seen a marked improvement take place in some cases when a stimulant has been left off. It is also a fact, that in bronchitis I have repeatedly seen improvement after stimulants have been omitted; and as regards heart-disease, I am convinced that the amount of mischief done by stimulants is immense. In the case of fevers and bronchitis, the weak pulse is often but an indication of extreme capillary congestion, and a stimulus to the heart only aggravates the evil. And in the case of a diseased and weak heart, where repose is indicated, a constant stimulation by alcohol adds immensely to the trouble." Dr. Wilks also refers to the common practice of advising "a little" alcohol, without defining the precise amount—"a little" generally meaning just as much as the patient chooses to take. He says with great propriety, that it is as important to define the quantity of this article as of opium or any other medicine. Abused as alcoholic stimulants are, by many American physicians, it is evident that British practitioners are much more reckless and undiscriminating in their applications. It must be so, to draw out from Dr. Wilks such a remark as this: "I do speak strongly against this indiscriminate use of alcohol in disease, without due consideration of its need or of its results."—Perhaps the late Dr. Todd is more responsible than any other individual for the reckless and indiscriminate use referred to. The practice has been further extentended by the doctrine that disease is always weakness, as advocated by Chambers and others. We may infer the extent to which the profession in England is given to the practice of alcoholic medication, by the following curious passage, taken from the lecture before us: "I do not wish to speak too dogmatically of its ill effects, being fully aware that there are many holding very distinguished positions in the profession whose opinions are not in accordance with those I have expressed. Were it not for this reason, I should have used still stronger language than I have done; for even firm convictions must be restrained when we know what an amount of strong opinion can be arrayed against us."—An extraordinary statement this—scarcely creditable to a manly and independent mind. But what must be the status of the profession in Great Britain on this question, when the discovery begins to be made that alcoholic stimulants are not an absolute necessity in fevers, and when a distinguished physician feels constrained to apologize for such sentiments as are quoted above, and to withhold the free utterance of his conviction!—*Pacific Med. & Surg. Jour.*

